# Some Problems Solved*This paper was received too late to appear under its proper head of Original Communications.—Editor.

**Published:** 1898-08

**Authors:** R. W. Lowe

**Affiliations:** Ridgefield, Conn.


For the Texas Medical Journal.

### Some Problems Solved. *

BY R. W. LOWE, M. D., RIDGEFIELD, CONN.

Problems are constantly presenting themselves to the busy practitioner, for solution. Some are knotty and difficult of solving so as to be acceptable to himself and to the profession, and render a cure possible, the result satisfactory to the patient. Those of diagnosis come first and of treatment afterwards.

The doctor's means of existence, the necessities for himself and family depend on the skill with which he shall relieve suffering and cure disease.

If he does this fairly well a living, at least, is for him, possi-

*This paper was received too late to appear under its proper head of Original Communications.—EDITOR.]

bly a competency, and if brilliant, wealth. These problems present themselves to all classes of practitioners, the country and city alike, as well as the rich and the poor, the talened and the ordinary.

After a considerable experience in the practice of my profession, and coming in contact socially and professionally with many of its brightest lights, I am forced to confess that they all have these problems to solve, to accomplish which they have quite as much difficulty as their more modest neighbor, the country doctor, with honors quite easy as to possible errors of judgment.

It is a good thing that we do not any of us know it all, for it is just such problems that stimulate us to higher endeavor and make honorable competition possible, something to work for besides the constant struggle for the almighty dollar.

The following case, which indirectly led to the treatment adopted in the other two, was just such a problem, to solve which, necessitated a considerable amount of study on my part, and the solution when arrived at, so impressed me that I considered it a duty to have it published, for the lesson there learned may tend to throw light upon some case which now rests in obscurity.

Mr. M., a banker, American, 66 years old, married, a man of family, consulted me on the 6th day of March, 1898, and I obtained from him the following facts: His family history was excellent, both parents dying at a good old age, the father at 77, and the mother at 83. He had one brother and two sisters, all married, who had raised families and were still living. He was born in the country, educated there and entered the office of a small private banking house in a neighboring city at the age of 22. His habits were exemplary, was a member of the church and Sunday school, never drank except in the most moderate way and at the table socially. Never drank in his life to excess. He did not smoke, and a very careful examination failed to reveal any specific lesion or history. He had married at 26 a lovely woman, who bore him three healthy children, and who made his home an exceptionally happy one. At the time of his marriage he moved to New York City and was engaged in a large banking house, of which he ultimately became the head. He was wrapped up in his business and the accumulation of wealth, while outside of this he cared for little besides his family, of whom he was inordinately fond. His habits were sedentary, taking but little of that exercise out of doors which is so essential to health.

He was five feet, nine inches tall and weighed 180 pounds, not what yon might call a fat man, but one simply showing good keeping. His appetite was always good, preferring plain food and cooking, meat forming a large part of his diet. At the age of thirty-six he commenced to have attacks of headache which lasted from that time till now, a matter of thirty years. These headaches were of a peculiar character.

The afternoon before the attack would come on he would be restless and uneasy, no pain, no indigestion, but a simple nervousness, a cloud would rise on the horizon betokening (as he often said) the coming of the headache storm of the next day. The pain would commence at the back of the head at the base of the brain, gradually increasing till the whole organ was one aching, throbbing mass. The sight was dim, conjunctiva congested, but the pupils showed no change.

Finally he was compelled to go to bed, there to remain for a day at least, and many times two. When he left it, he looked as if he had been through a fit of sickness. Of course it is not necessary for me to say that with his means he could and did employ the best medical talent, in fact, both at home and abroad, he had the best that could be procured. He was medicined, shocked, douched, sprayed, physicked, injected and purged, anodyned and narcotized till, as he said, "it was no use," he had simply to live with it till he died or it killed him. These attacks at first came on about once a month, but as he grew older, they increased in frequency till at the time of my visit they came on once in about four days.

I was called to see him during an attack at about ten o'clock in the morning of the day in question. The room was darkened, perfect quiet pervaded the house from top to bottom, the servants going about on tip-toe. In fact everything tended to show that severe illness was present.

I found a slight rise in temperature of about one-half of a degree. The tongue, while not coated, was white and flabby, indicating an impaired digestion. His pulse was slightly accelerated, 88. He lay with his knee drawn up and perfectly quiet.

The lines of his face were drawn sharply, showing how intense his suffering was. His wife informed me that twice during these attacks lately he had had a mild delirium.

On questioning him about the condition of his bowels he said that they were very regular, and had moved the evening before. While the movements had been regular, he said that he had not for years felt the relief he should have done after a normal passage. He insisted that I should give him something simply to relieve the pain, nothing else, in fact. I was informed by him that I need not ask him questions, I was not called in for that. Simply relieve the pain by a hypodermic injection, and go away. If I did not do this, I could go away, as that was all I was called for. It had come at last to the only thing that was sure relief, the deadly morphine.

The case was an interesting one, and I sparred for time and wind.

I absolutely refused to give the morphine unless I had tried other means and found that they were of no avail. After a careful examination of his heart, and finding that all right, I administered a small quantity of chloroform by inhalation. This gave him some relief and a little sleep. While he was asleep I examined his urine, and found that it was acid, containing a quantity of phosphates above the normal, while the specific gravity was 1025.

A half hour's sleep brought him to consciousness and pain again.

I had in the interval made up my mind that it would do no harm and possibly some good if I unloaded his bowels thoroughly, so I gave him a teaspoonful of thialion dissolved in a teacupful of hot water and had him drink it as warm as possible. This was repeated in two hours. An hour after the last dose he had two copious evacuations, the large mushy stools, that thialon invariably produces.

While this medicine was getting in its fine work I had administered to him small quantities of chloroform so as to keep him quiet, but after the last passage he went to sleep and slept well till morning. I stayed all night with him.

In the morning I had a further talk with him which resulted in his consenting to take another dose of thialion which resulted during the day in two more passages and a copious flow of urine.

The relief that this thorough cleansing give him was immense and after my call at 6 p. m., I left him at his request for good.

Four days after my last visit he called at my office stating that he wanted to know what that medicine was I gave him and to get some more. He said it certainly made him feel better. I ordered him to procure a bottle of thialion and take a teaspoonful each morning on rising dissolved in the same quanity of hot water.

It was to be taken as hot and as early after waking as possible. He called again in a week and to my surprise, he said that he had not had any headache since I made my first visit, but he felt a little nervous and was afraid he would have one in the morning and asked me to be sure and call, which I did. I found him with one of the headaches sure enough, but of a very mild character. By ten o'clock he was out doors walking about, a thing he had not done in years on a headache day.

According to my instructions he continued the thialion every morning and from this time on for the next six weeks he had two mild attacks only and then they left him for good, at least he has not had an attack since. He still takes a teaspoonful of thialion twice and sometimes three times a week.

I did not at my first visit think that case was one of uric acid headaches. In fact I was at a loss for a diagnosis and I gave the thialion alone to relieve the hepatic torpor and sluggishness of the bowels that I felt sure to exist. The effect of the remedy was so marked from the very first almost, and its continuance so happy in results that I am satisfied that the uric acid played an important part in this drama of pain and suffering and that the thialion united with the uric acid forming a soluble salt which was quickly washed out of the system and the cause of all his sickness removed. It is unncessary for me to say that Mr. M. is my good friend and he showed his appreciation of my skill in getting him out of his trouble with a handsome check.

Case 2 was that of Mrs. C., aged 59 years, who weighed 190 pounds. She was wealthy, a high liver, meat forming a prominent part of her diet. I was called to see her early in the morning of April 7th and found her suffering from a severe attack of neuralgia of the right arm. The pain was so lancinating and so severe as to necessitate a hypodermic injection at once and before I could make an extended examination. After it had ceased, she told me she had suffered from this pain at intervals differing as to the length of time for the last three years. She had tried a host of remedies and doctors, but without any permanent benefit, the morphine alone giving relief.

This had to be continued for a couple of days when the pain went away only to return again in a short time.

Having the case of Mr. M. in mind, gave her a teaspoonful of thialion every two hours till the bowels moved thoroughly, which happened after the fourth dose, the morphine probably interfering with the action of the medicine somewhat. She, as well as myself, was astonished, when at my evening visit she informed me that she had had no more pain and that she was quite comfortable, and I found the same condition in the morning when I made my next visit.

I directed that the thialion should be kept up in the same dose and manner twice a day for two days, and then but once a day, that in the morning. In the course of two weeks, there being no return of the pain, the dose of thialion was taken but twice a week. From that time till now she has had no return.

The neuralgic pain in this case was without doubt due to the excess of uric acid in the system and which the thialion dissolved, freeing her from her enemy of years standing.

Case 3. M. D., a mechanic, called at my office on the 2d day of May, giving me the following history. He was 29 years old, American, married, man of family, temperate, but using tobacco freely. A hearty liver, weighing about 170 pounds and standing about six feet in his stockings. His bowels were regular, but for the last three months he had suffered very badly with irritability ofthe bladder. He could not hold his water as he used to do, and made it five or six times a day, and what was the most annoying of all he had to get up two or three times in the night.

He said he had had no blow or other cause that he knew of to give rise to the trouble, but it was mighty inconvenient, and he wanted it stopped. Knowing from experience that thialion will remove pain in the kidneys when present from irritation, I thought I would try it on him. I gave him a prescription calling for a bottle, directing him to take a teaspoonful dissolved in a teacup of hot water three times a day, and to come and see me on the second day after. He did this, and on his next visit he complained of a very bad looseness of his bowels, but his pain was much better.

I directed him to take the thialion every morning on rising, and in a week he was relieved from all of his disagreeable symptoms. The third day he stopped me while I was passing his house, saying he wanted to show me what he passed with his water. At the bottom of the vessel which he had saved there was a thick coat of uric acid crystals. They were fastened to it, and could hardly be removed with sapolio. The quantity was greater than I ever saw in any specimen before.

This case was a revelation to me of the power of this drug, and its wide range of application.

